# Protein Complexes Prediction Method Based on Core—Attachment Structure and Functional Annotations

**DOI:** 10.3390/ijms18091910

**Published:** 2017-09-06

**Authors:** Bo Li, Bo Liao

**Affiliations:** College of Computer Science and Electronic Engineering, Hunan University, Changsha 410082, China; dragonbw@163.com

**Keywords:** protein–protein interaction network, overlapping, clustering

## Abstract

Recent advances in high-throughput laboratory techniques captured large-scale protein–protein interaction (PPI) data, making it possible to create a detailed map of protein interaction networks, and thus enable us to detect protein complexes from these PPI networks. However, most of the current state-of-the-art studies still have some problems, for instance, incapability of identifying overlapping clusters, without considering the inherent organization within protein complexes, and overlooking the biological meaning of complexes. Therefore, we present a novel overlapping protein complexes prediction method based on core–attachment structure and function annotations (CFOCM), which performs in two stages: first, it detects protein complex cores with the maximum value of our defined cluster closeness function, in which the proteins are also closely related to at least one common function. Then it appends attach proteins into these detected cores to form the returned complexes. For performance evaluation, CFOCM and six classical methods have been used to identify protein complexes on three different yeast PPI networks, and three sets of real complexes including the Munich Information Center for Protein Sequences (MIPS), the Saccharomyces Genome Database (SGD) and the Catalogues of Yeast protein Complexes (CYC2008) are selected as benchmark sets, and the results show that CFOCM is indeed effective and robust for achieving the highest F-measure values in all tests.

## 1. Introduction

Most proteins in living organisms, performing their biological functions or involving with cellular processes, barely serve as single isolated entities, but rather via molecular interactions with other partners to form complexes [1]. In fact, protein complexes are the key molecular entities to perform cellular functions, such as signal transduction, post-translational modification, DNA transcription, and mRNA translation. Moreover, the damage of protein complexes is one of the main factors inducing severe diseases [2]. Identification of protein complexes, therefore, becomes a fundamental task in better understanding the biological functions in different cellular systems, uncovering regularities of cellular activities and contributing to interpreting the causes, diagnosis, and even the treatments of complex diseases. As a result, lots of techniques including laboratory-based and computational-based have been proposed to address this issue.

Up to now, significant progress in high-throughput laboratory techniques involving Tandem Affinity Purification (TAP) [3] and Mass Spectrometry (MS) [4] has been made to discover protein complexes on a large scale. However, laboratory experiments are expensive and time-consuming, resulting in poor coverage of the complete protein complexes. Fortunately, the genomic-scale protein–protein interaction (PPI) networks created from pairwise protein–protein interactions make it possible to automatically and computationally detect protein complexes. Given a PPI network, as the protein complexes are formed by physical aggregations of several binding proteins, they are assumed to be the functionally and structurally cohesive substructures, and thus graph clustering methods have been put forward to search densely connected regions in PPI networks as protein complexes.

Since some proteins have multiple functions, in other words, they may belong to more than one protein complex, so the ideal approaches need to be able to detect overlapping complexes. However, several types of graph clustering methods don’t allow overlaps between detected protein complexes due to the confinements of the rationales behind them. For example, the partition-based clustering methods such as the Restricted Neighborhood Search Clustering algorithm (RNSC) [5], the Bayesian Nonnegative Matrix Factorization(NMF)-based weighted Ensemble Clustering algorithm (EC-BNMF) [6], obtain, however, some highly reliable protein complexes, since they need prior knowledge of the exact number of clusters that thus cannot detect overlapping functional modules, and, in addition, most of the hierarchy-based clustering methods [7,8,9] utilize hierarchical trees to represent the hierarchical module organization for a PPI network, but it is difficult to detect overlapping complexes as well. In addition, although some algorithms are capable of finding overlapping complexes, they still have some distinct shortcomings—for instance, the Molecular Complex Detection (MCODE) [10] predicts only quite a small number of protein complexes. CFinder [11] first discovers k-cliques by using the clique percolation method (CPM) [12], and then combines the adjacent k-cliques to get the functional modules, but may fail to detect some regular complexes. ClusterONE [13] requires one pre-determined parameter, which is depended on the quality of PPI network, and it is difficult to determine.

Furthermore, the aforementioned methods still have a common fatal weakness—ignorance of the inherent organization of the complexes—but actually experimental analysis has already reported that a protein complex generally consists of a core, in which proteins share similar functions and tend to be highly co-expressed, and other attach proteins surrounding to the core [14]. Based on these, several core–attachment based algorithms have been presented, and experimental results indicate that they can acquire better performance compared to traditional methods neglecting inherent organization. Among them, CORE [15] first calculates the probability of each pairwise proteins to be in the same core and then uses it to detect cores. COACH [16] detects cores from neighborhood graphs of the selected seed proteins, and then applies an outward growing strategy to generate protein complexes. Compared with CORE, COACH can find overlapping cores. Other methods including [17] predict complexes based on multi-structures in PPI network, and achieve significant performance. The complexes predicted by structure-based methods, in general, have been verified more in accordance with the known complexes.

In addition, to precisely predict more biological explainable complexes, some methods of fusing various types of prior knowledge including functional annotations [18,19,20], gene expression data [21,22,23], as well as sub-cellular location of proteins [24], are presented and have already been proved that can help to improve the performance to some extent. However, these kinds of valuable information are either used in data preprocessing or post-processing, such as filtering low-confidence edges, weighting edges, discarding some biological meaningless complexes, but seldom helps mining cores with better biological meaning, in which most proteins are co-subcellular or co-expression or with similar functions. Furthermore, since these data are undeniably incomplete and imprecise, how to generate a impartial and efficient model incorporating different types of data is still a hot topic in complex prediction [25,26,27].

In summary, we may come to the conclusion that a comparatively well-designed protein complexes identification method may need to meet the following conditions: capable of detecting overlapping complexes, fewer parameters, being easy to be determine, consideration of the inherent organization of protein complexes, particularly finding topological and biological meaningful cores, properly incorporating prior information as much as possible into the predicting model, and robust to PPI networks with false positives and false negatives. Unfortunately, even though many effective techniques have been proposed, as far as we know, few of them satisfy most of the above-mentioned requirements, which results in impeding further practical applications, and thus there is still urgent need for new approaches.

In this manuscript, we introduced a novel core–attachment based method to predict protein complexes, and the proteins in our detected cores are closely linked, share high similar topology that is highly connected to internal vertexes and relatively sparsely connected to outsides, and are more biologically significant, namely more likely to participate in one or more biological processes with the appliance of GO functional annotation. Furthermore, the detected complexes can be overlapping. We applied our algorithm to two PPI networks of yeast, and validated our predicted complexes using benchmark complexes collected from several public databases. The experimental results indicated that our algorithm is efficient and outperforms other existing classical methods.

## 2. Results

We have applied our CFOCM method on the Database of Interacting Proteins (DIP) data and Gavin data. In this section, we will first discuss parameter *t* affecting the performance of CFOCM. Next, we perform comprehensive comparisons with various existing classical methods and analyse the results in detail. Finally, we explore the functional definition of the complex-core as a whole, contributing to the biological significance of the detected complexes.

### 2.1. Evaluation Metrics

The neighborhood affinity score NS(p,r) can also be devoted to measure the overall similarity between a predicted complex *p* and a real complex *r*, and if NS(p,r)≥ω, *p* and *r* are considered to be matching. On the one hand, the greater setting value of ω means the more stringent matching of between the predicted complex and the real complex in the benchmark, probably resulting in a sharp decline in all the prediction measure values; on the other, the smaller value could not only lead to identify the low-confidence predicted complex as the real complex, which is also not reasonable. In our experiments, we set ω to 0.2 the same as most literatures do [5,7,11,13,15,28], which provides easy and fair comparisons between results of various algorithms.

Let *P* and *R* represent the set of predicted complexes and the real complexes in benchmarks, respectively. $N_{cp}=\left\{p\in P|\exists r\in B,\mathrm{NS}\left(p,r\right)\geq \omega \right\}$ denotes the predicted complexes matching at least one real complex, and $N_{cr}=\left\{r\in R|\exists p\in P,\mathrm{NS}\left(r,p\right)\geq \omega \right\}$ denotes the real complexes matching at least one predicted complex. In addition, then the performance of a clustering algorithm can be measured using precision, recall, and F-measure, which can be calculated as follows:

$$\mathrm{Precison}=\frac{|N_{cp}|}{\left|P\right|}\;\;,$$

$$\mathrm{Recall}=\frac{|N_{cr}|}{\left|R\right|}\;\;,$$

F-measure=2×Precison×Recall/(Precision+Recall),

where Precision means the ratio of predicted protein complexes that are matched with the real complexes, Recall means the rate of real complexes that are successfully detected and F-measure evaluates the overall performance.

### 2.2. Optimization of the Parameter t

**Figure 1:**
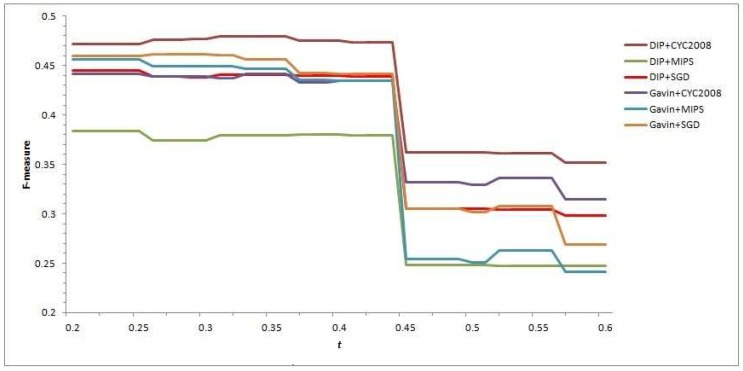
The effect of *t*, showing how the variation of parameter *t* affects the performance of our proposed overlapping protein complexes prediction method based on core–attachment structure and function annotations (CFOCM) in terms of F-measure.

Recall that the process of mining cores from PPI network in Algorithm 1 of CFOCM employs a user-defined parameter *t* calculated by $\mathrm{NS}(\;mc_{i},mc_{j})$ to decide whether a certain candidate core $mc_{j}$ should be merged into the family of the current candidate core $mc_{i}$. In general, CFOCM can predict more complexes with the bigger value of *t*; nevertheless, this may lead to compromise on the quality of the predicted complexes, and thus how to choose a relatively appropriate *t* to achieve a balance between the predicted complexes’ quality and quantity needs to be probed. Here, varying *t* from 0.2 to 0.6 with the interval 0.01, the F-measure values of each predicted complex set are computed, and help us to intuitively observe that the variation of *t* affects the performance of our CFOCM method and selects the relatively suitable *t* as well (see Figure 1).

In Figure 1, all the curves of different CFOCMs, based on DIP data or Gavin data, validated in benchmark set MIPS or SGD or CYC2008, are comparably smooth and steady when the *t* varies from 0.2 to 0.44. However, the curves change abruptly near t=0.45, and the causation of this phenomenon can be rationally explained with the NS score of two candidate cores being 4/9 (≈0.44) in which the number of proteins are both three and two of them are the overlapping; that is to say, these two cores can not be put into the same family if *t* is larger than 4/9, resulting in a rapid increase of low-confidence detected cores with size 3 and a sharp decease of recall value and F-measure score as well. For example, under t=0.44, CFOCM based on DIP and Gavin network generates 751,453 complexes respectively, while under t=0.45 generates 2629, 1703 complexes respectively, conforming to the above analysis and interpretation.

As stated above, *t* should definitely not be set to larger than 0.44 as increasing abundant low-confidence three-size cores, and actually the performance of CFOCM does not change significantly when t∈[0.2,0.44]. Still, demand for more complexes shows a preference to a larger *t*; otherwise, if there is demand for a fewer number of complexes, a preference is shown for a smaller *t*. For example, CFOCM predicts 545 complexes with average matching of 156 real complexes in MIPS when t=0.2, while predicting 751 complexes matching 205 real complexes in MIPS when t=0.44. In the following part, either in DIP data or Gavin data, the *t* of our CFOCM algorithm is set to 0.4.

### 2.3. Comparison Experiments on Different Datasets

**Figure 2:**
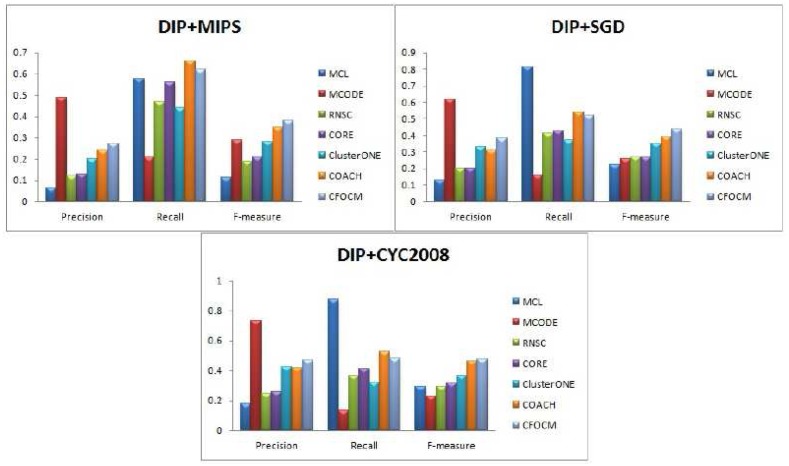
Comparative performance of CFOCM and the other six methods in DIP data using benchmark MIPS, SGD, CYC2008, respectively.

**Figure 3:**
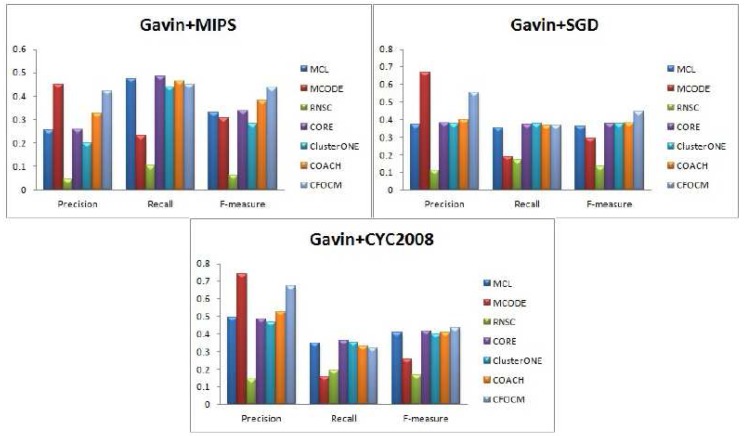
Comparative performance of CFOCM and the other six methods in Gavin data using benchmarks MIPS, SGD, CYC2008, respectively.

**Figure 4:**
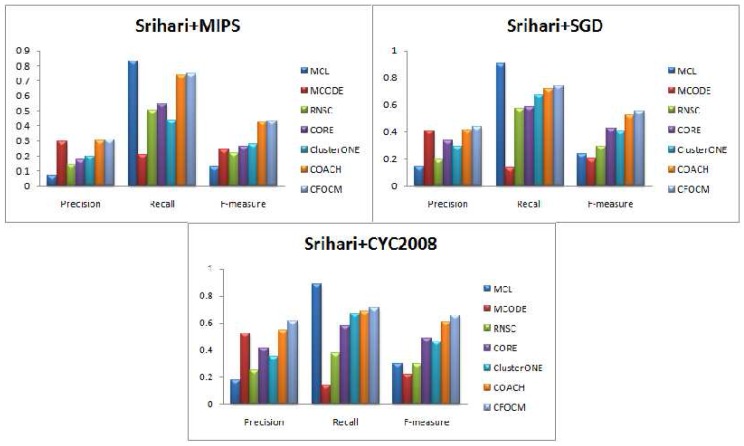
Comparative performance of CFOCM and the other six methods in Srihari data using benchmarks MIPS, SGD, CYC2008, respectively.

For performance evaluation, the comparison experiments between CFOCM and six representative algorithms including MCL, MCODE, RNSC, CORE, COACH and ClusterONE are performed on both DIP data , Gavin data and Srihari data. Note that the parameters of these six comparative methods are set to the default values. Figure 2, Table 1, Figure 3, Table 2 ,Figure 4, and Table 3 exhibit the overall comparison results in terms of Precision, Recall and F-measure on DIP data, Gavin data and Srihari data, respectively.

In Figure 2, no matter whether benchmarks MIPS or SGD or CASP2008 are used, MCODE achieves the highest precision that is far beyond other methods. However, since the number of predicted protein complexes is very limited and also matches with fewer real complexes, resulting in much low recall and F-measure values. In addition, CORE, RNSC, and ClusterONE are observed to attain high recall values, but, nevertheless, the F-measure values of them merely end up with relatively lower F-measure value due to their very low precision values. In fact, CFOCM and COACH demonstrate their distinctive competitive advantages in F-measure as a result of balanced precisions and recalls. Moreover, it is obvious that CFOCM remarkably outperforms COACH in F-measure when using benchmark MIPS and SGD. Meanwhile, both CFOCM and COACH are based on core–attach structure, it may indicate that the protein complex detection method seems more appropriate when taking consideration of the inherent organization of complex. As Table 1 shows, CFOCM detects moderate number of complexes, many of which correctly match with the real complexes and have a high coverage rate of real complexes as well.

In order to evaluate the robustness of algorithm CFOCM, comparison experiments are also carried on Gavin network, which is different from the DIP network for containing much fewer and more densely connected proteins. Figure 3 illustrates the results for Gavin data, CFOCM shows even better performance for Gavin data, which achieves the highest precision values when using benchmark MIPS and CYC2008, and, apparently, CFOCM obtains the best F-measure value for every benchmark. This may suggest that CFOCM indeed works on dense network as well. For each method, the total number of identified complexes, the number of correct predictions $N_{p}$ matching at least a real complex, and the number of real complexes $N_{b}$ matching at least a predicted one are listed in Table 2, reaching similar conclusions that are consistent with DIP data.

For further evaluation, Srihari data derived from three different repositories are also used for comparison, and the results are showed in Figure 4 and Table 3. Similar conclusions can be reached as in DIP and Gavin data, except that both the Precision value and Recall value of CFOCM are better than COACH, and this may indicate that CFOCM has more potential on composite data.

In a word, either in relatively sparse DIP networks or in relatively dense Gavin data even using a composite data set, CFOCM is able to identify a suitable number of protein complexes, and, meanwhile, the predicted complexes are also biologically meaningful as a consequence of cooperating the protein function annotations into our model, so it compellingly performs better than other existing methods in term of F-measure. Thus, we can come to the conclusion that CFOCM is efficient and has strong adaptability and robustness to different types of data.

## 3. Discussion

### 3.1. The Effectiveness of Functional Annotation

As the assumption of the complex-core described before, the proteins in each CFOCM detected core must be functional related to a certain common GO item, namely either annotated with that GO item or annotated with a GO item that is functionally interdependent with that GO item. To estimate the contribution of this, comparison experiments between CFOCM and CFOCM without use (unCFOCM) are conducted. As the results listed in Table 4 (DIP) and Table 5 (Gavin), unCFOCM in all the tests predicts much more biological meaningless complexes on account of not using GO annotation, leading to lower F-measure values. In other words, owing to the requirement of functional relevance within the discovered cores, CFOCM is capable of filtering abundant low-confidence protein complexes, and the detected protein complexes are supposed to be more biologically significant. Therefore, the cores detected by CFOCM should share some common functions, which is more in conformity with the original definition of the complex core, and it is greatly obliged to help finding more accurate protein complexes.

### 3.2. Case Studies

**Figure 5:**
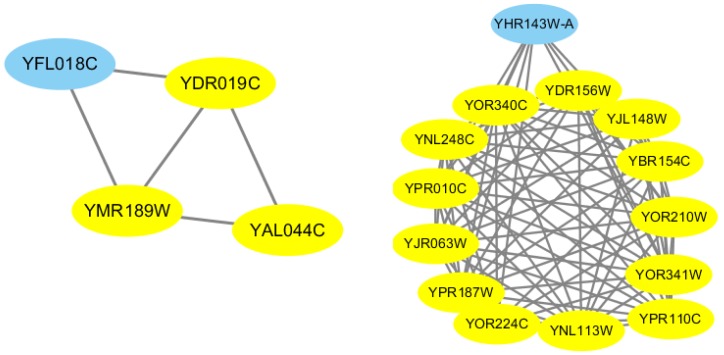
The Glycine decarboxylase complex and the RNA polymerase I complex as detected by CFOCM. The yellow nodes represent proteins within the complex core, while the blue node proteins represent proteins that are attachments.

This section illustrates two predicted protein complexes, namely the Glycine decarboxylase complex and the RNA polymerase I complex as Figure 5. The Glycine decarboxylase complex is a small-sized complex responsible for the oxidation of glycine by mitochondria, and it consists of four proteins including YDR019C, YMR18W, YAL044C and YFL018C. As showed, CFOCM successfully identified these four proteins, in which YDR019C, YMR18W, and YAL044C are recognized as core proteins and YFL018C is detected as an attachment to the core. In another case, the RNA polymerase I complex is a larger complex comprised of 14 proteins, and CFOCM could also completely identify all the proteins in this complex with 100% precision, in which all proteins except YHR143W-A are detected as members of the core having more dense connections with each other and sharing more functional relevance as well.

## 4. Materials and Methods

### 4.1. Terminologies

A PPI Network typically can be represented as an undirected graph G=(V,E), where *V* and E={(u,v)|u,v∈V) represent proteins and protein–protein interactions, respectively. A graph $G^{\prime }=\left(V^{\prime },E^{\prime }\right)$ is regarded as a subgraph of *G* if $V^{\prime }\subseteq V\;\;\;\;\mathrm{and}\;\;\;\;E^{\prime }\subseteq E$. *v*’s direct interacting neighbors in graph *G* is denoted as $N_{v}=\left\{u|\left(u,v\right)\in E,\;\;\;\;u\in V\right\}$, and $N_{v}^{G^{\prime }}=\left\{u|\left(u,v\right)\in E,\;\;\;\;u\in V^{\prime }\right\}$ is *v*’s neighbors in subgraph $G^{\prime }$. Subgraph $G^{\prime }$ external boundary nodes are defined as $V_{ob}\left(G^{\prime }\right)=${v|<v,w>∈E(G),v∈$V\left(G\right)\setminus V\left(G^{\prime }\right)\;\;,w\in V\left(G^{\prime }\right)\}$.

A neighborhood affinity score metric [25], denoted as $\mathrm{NS}(G^{\prime },G^{\prime \prime })$, is imported to measure the similarity between two overlapping graphs $G^{\prime }=\left(V^{\prime },E^{\prime }\right)$ and $G^{\prime \prime }=\left(V^{\prime \prime },E^{\prime \prime }\right)$,

$$\mathrm{NS}\left(G^{\prime },G^{\prime \prime }\right)=\frac{|V_{G^{\prime }}\cap V_{G^{\prime \prime }}{|}^{2}}{|V_{G^{\prime }}\left|\times \right|V_{G^{\prime \prime }}|},$$

where, if $\mathrm{NS}(G^{\prime },G^{\prime \prime })>=t$ (*t* is a predefined threshold), we may declare cluster $G^{\prime }=\left(V^{\prime },E^{\prime }\right)$ and cluster $G^{\prime \prime }=\left(V^{\prime \prime },E^{\prime \prime }\right)$ can be further merged as a result of their high topological similarity.

As is well known, GO is composed of three orthogonal ontologies capturing knowledge about biological process, molecular function and cellular component, and each ontology consists of controlled and structured biological terms that can be used to annotate genes and proteins. Some GO item pairs are highly functionally related—for example, sharing a common parent node, or one is just a near ancestor of the other, while other GO item pairs may possess much weaker relationships or even be functionally independent. Therefore, the urgent need is to design a metric to quantify the functional interdependence between two GO items. Fortunately, Ref. [18] has done what we want (see the formula below):

$$fr_{i,j}=\frac{re_{i,j}-ee_{i,j}}{\sqrt{ee_{i,j}(1-(\sum_{k\in GI}ee_{i,k}/|E|))(1-(\sum_{k\in GI}ee_{k,j}/\left|E|)\right)}},$$

where $re_{i,j}$ represents the real number of edges in *G* connecting one protein annotated with GO item *i* and the other annotated with item *j*, $ee_{i,j}$ represents the expected number of edges that one protein is annotated with item *i* and the other annotated with item *j* in *G*, hence it equals (Number of edges in *G* with one protein annotated with *i*)*(Number of edges in *G* with one protein annotated with *j* to the others)/|E|, and GI represents the whole GO items set. Ref. [18] also indicates that item *i* and *j* are functionally interdependent if $fr_{i,j}>1.96$; otherwise, they are considered to be functionally independent.

A protein complex is pervasively modeled as an induced subgraph of PPI network *G*, the proteins in which have dense intra-connections and are sparely connected to the rest of the network, thus we introduce a new and effective closeness function to quantify the probability that $G^{\prime }$ is complex based on network topology:

$$\mathrm{cf}\left(G^{\prime }\right)=\mathrm{density}\left(G^{\prime }\right)\times \left(\frac{1}{|G^{\prime }|}*\sum_{v\in G^{\prime }}\frac{|N_{v}^{G^{\prime }}|}{|N_{v}|}\right),$$

where $\mathrm{density}\left(G^{\prime }\right)=\frac{2\times |E^{\prime }|}{|V^{\prime }|\times (|V^{\prime }\left|-1\right)}$ is the density of graph $G^{\prime }$, depicted to quantify the richness of edges in $G^{\prime }$, and $\frac{|N_{v}^{G^{\prime }}|}{|N_{v}|}$ corresponds to the percentage of *v*’s direct neighbors located within $G^{\prime }$. If $\frac{|N_{v}^{G^{\prime }}|}{|N_{v}|}$ equals 1, all the neighbors of *v* are in $G^{\prime }$, so there is a high tendency that *v* should be a member of $G^{\prime }$. If equals 0, *v* has little chance to be a member of $G^{\prime }$. Consequently, the expression in the bracket represents the mean possibility of each node being retained in $G^{\prime }$. Compared with previous closeness function based on the density of $G^{\prime }$, cf not only assesses the inner denseness of $G^{\prime }$, but also takes the ratio of $G^{\prime }$ inner edges and outer edges into consideration, hence manifesting superiority in appraising the likelihood of $G^{\prime }$ to be a real complex.

### 4.2. Description of CFOCM Algorithm

Most of the protein complexes contain core–attachment structure, and the proteins in the core share similar topology and are highly functionally related, while the attach proteins are usually located in the periphery of the core [14]. As the differences between core proteins and attach proteins, therefore, our core–attachment based algorithm CFOCM for protein complexes identification, comprised of two necessary phases, which first detects the protein complexes’ cores and then selects attach proteins to the discovered cores.

### 4.2.1. The Complex Cores Detection

Protein-complex core plays a key role for complex performing biological function, and determines the cellular role and significance of the complex in the context to a large extent [14]. The results of biological analysis also indicate that most protein complex cores own some significant distinguishing features: including a small group of proteins which are densely intra-connected and sparsely to outsides, allowing overlaps between cores, possession of some common functions, showing an altitudinal mRNA co-expression patterns. In this paper, however, only the former three features are used to portray the cores discovered by CFOCM, and our detected cores satisfy the following assumption.

**Assumption** **1.** *A subgraph $G^{\prime }=\left(V^{\prime },E^{\prime }\right)$ is a protein-complex core unless if satisfying the followed conditions:*

*1*. The topology of $G^{\prime }$ meets: $|G^{\prime }|>=3$, $G^{\prime }$ reaches the local optimum that there does not exist any neighbor node v that satisfies $\mathrm{cf}\left(G^{\prime }+\left\{v\right\}\right)>\mathrm{cf}\left(G^{\prime }\right)$ or $\mathrm{cf}\left(G^{\prime }-\left\{v\right\}\right)>\mathrm{cf}\left(G^{\prime }\right)$, and no such $G^{\prime \prime }$ exists if $G^{\prime }\subseteq G^{\prime \prime }\;\;\;$ and $G^{\prime \prime }$ is a complex core.
*2*. If $G^{\prime }$ has overlaps with $G^{\prime \prime }$, then $\mathrm{NS}(G^{\prime },G^{\prime \prime })<t$ must be satisfied; otherwise, $G^{\prime }$ and $G^{\prime \prime }$ could combine together.
*3*. $G^{\prime }$ needs to be biologically significant: mx is defined as the the maximum common GO item annotating a maximum number of nodes in $G^{\prime }$, $\forall v\in V^{\prime }$, v is either annotated by mx or annotated by a GO item gi interdependent with mx, which satisfies fr(gi,mx)>1.96.

Different from traditional methods exploring each core protein separately, our above complex-core assumption is more plausible for considering all proteins in the core as a whole. Benefiting from this renovation ensures that each protein in the core owns similar topology and contributes to the enforcement of core’s biological functions. Conditions 1, 2, and 3 guarantees the maximizes closeness function value of core, the nearest distance can be retained between different cores, and participation of at least one common biological functions, respectively. Specifically, most traditional literature is mainly focused on the assurance of highly functional similarity between each protein pair in the core, which will result in neglecting that the core as a whole should perform some common functions, while this flaw is certainly renovated by our integrated global view of the core.

Algorithm 1 illustrates that the overall framework to detect protein-complex cores, and, without question, the discovered cores comply with definitions in Assumption 1. We first compute the functional interdependence between each GO items pair by the definition fr in line 1. Then, in line 2, we identify all cliques that are fully connected subgraphs by using a complete enumeration method [29], based on the fact that a k-clique can be obtained by adding a vertex to the clique with k-1 vertices and the 2-cliques can be initialized as the edges in the graph, but only the maximal cliques are reserved at last, and a k-clique is regarded as a maximal k-clique only in the case that it cannot be enlarged by adding any vertex. After that, lines 4–19 mining complex cores by a iteration process on the basis of the two aforementioned pretreatment works. Here, a concept of candidate-core family is presented, containing the core itself and its similar candidate-cores with the neighborhood affinity score NS less than a predefined threshold *t*. For each certain candidate-core, its family set is obtained in lines 8–13, and a more reasonable combined candidate-core comes into being through algorithm Merge_Similar_Cores in line 14. The details of Merge_Similar_Cores algorithm are described in Algorithm 2. Still, in lines 15–17, if the current generated candidate-core already exists in the generated candidate-core set, we simply discard it; otherwise, we add it to the candidate-core set. After these steps, though, there unavoidably exist some incorrect manipulations, excessive overlapping and biological meaningless candidate cores are substantially removed, and the overwhelming majority of the vertexes in retained cores are densely connected internally, possess similar topology and attend to share at least one common GO annotated function.

**Algorithm 1:** Complex cores detection algorithm.**Require:** The PPI network G=(V,E);   Neighborhood affinity score threshold *t*. **Ensure:** The detected complex cores set CS. 1:calculate each GO item pair functional interdependence fr;2:find all the maximum cliques MC in *G*;3:CS=MC;4:**repeat**5: MC=CS;6: CS={};7: **for** $mc_{i}$ in MC **do**8:  $F_{mc_{i}}=\left\{\;mc_{i}\right\};$ {$F_{mc_{i}}$ stores the cliques similar with $mc_{i}$}9:  **for** $mc_{j}$ in MC **do**10:   **if** $\mathrm{NS}(\;mc_{i},mc_{j})>=t$ **then**11:    $F_{mc_{i}}=F_{mc_{i}}\cup \left\{mc_{j}\right\};$12:   **end if**13:  **end for**14:  $c=Merge\_Similar\_Cores(F_{mc_{i}});$
15:  **if** *c* is not exists in CS **then**16:   CS=CS∪{c};17:  **end if**18: **end for**19:**until** not exists any two elements $c{}_{i}$ and $c{}_{j}$ in CS satisfying $\mathrm{NS}(c_{i},c_{j})>=t$20:**return**CS;

A crucial artifice, not described in Algorithm 1, is applied in the process of detecting cores. First, for each maximal cliques set with the same number of vertexes, we generate their corresponding new candidate cores by executing steps in lines 4–19, and then form the final detected cores via the same steps on these different-sized generated cores. Without using this, the smaller cliques may be annexed by the larger similar cliques so that they barely contribute to the generation of the new candidate core. Actually, this artifice is proved to be an effective means of improving the predicting performance.

### 4.2.2. Similar Complex Cores Merge

Given the family $F_{mc}$ of the candidate core mc, the Merge_Similar_Cores algorithm will filter the proteins that can not help to preserve the topology of the core or are functionally independent with other proteins in the core and return a new candidate core.

Our Merge_Similar_Cores algorithm works as follows. To begin with, we extract the proteins PS from the input family of a candidate-core in line 1, and find the GO item *m* disappeared in the GO annotations of maximal proteins in line 2. Afterwards, in lines 3–7, we remove proteins that are neither annotated by the common item *m* nor have a GO item functional interdependent with item *m*, and this procedure ensures that the returned candidate-core has a high probability of owning at least one common GO function because the proteins in the returned candidate-core either have the common GO item *m* or a GO item *j* exists that is functionally interdependent with *m*. Finally, in lines 8–10, we iteratively delete a protein *p* from the PS until no such protein *p* exists, satisfying cf(PS-{p})>cf(PS), and ensuring that the remaining proteins reach the local optimum, which is relatively richly inner-connected and sparsely connected to the outside.    

**Figure 6:**
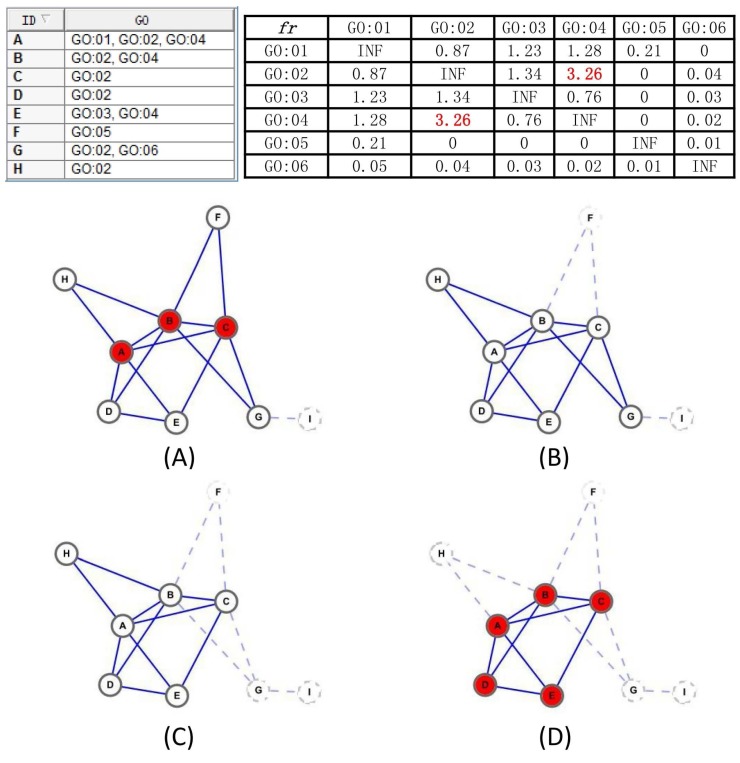
The diagram of Merge_Similar_Cores algorithm. In the example, (**A**) is the family graph of clique {A,B,C}, including cliques {{A,B,C},{A,B,D},{A,B,H},{A,C,E},{B,C,F},{B,C,G}}, and the proteins set is {A,B,C,D,E,F,G,H}. In (**B**), the common Gene Ontology (GO) item is GO:02, and reserve vertex E as $fr_{GO:02,GO:04}>1.96$, while drop vertex F is $fr_{GO:02,GO:05}<1.96$. In (**C**), drop vertex G is $\arg\max_{p\in PS}\mathrm{cf}\left(PS-\left\{p\right\}\right)=G$. In (**D**), drop vertex H is $\arg\max_{p\in PS}\mathrm{cf}\left(PS-\left\{p\right\}\right)=H$, and returns the next candidate-core A,B,C,D,E, as no remove operation can improve the cf.

**Algorithm 2: **c= Merge_Similar_Cores ($F_{mc}$).
1:get all proteins PS contained in $F_{mc}$;2:find the GO item *m* which annotating maximum number of proteins in PS;3:**for** each *p* in PS **do**4: **if** *p* is not annotated by *m* and exists no GO item *j* annotating *p* satisfying: $fr_{m,j}<1.96$ **then**5:  PS=PS-{p};
6: **end if**7:**end for**8:**while** exists $\max_{p\in PS}\mathrm{cf}\left(PS-\left\{p\right\}\right)>\mathrm{cf}\left(PS\right)$ **do**9: $PS=PS-\{\arg\max_{p\in PS}\mathrm{cf}\left(PS-\left\{p\right\}\right)\};$
10:**end while**11:**return** c=PS;


Each input candidate-core family goes through these steps, and a newer candidate-core has been formed. In addition, Figure 6 also provides an example to illustrate the process of our proposed Merge_Similar_Cores algorithm.

### 4.2.3. Attach-Proteins Screening

After the foregoing phase of our CFOCM method, the protein-complex cores have already been mined from PPI network G=(V,E). In the second phase, we will form the final predicted complex by appending reliable peripheral proteins to the discovered cores. Given a protein complex core *c*, for each external boundary protein *p* of current core *c*, the following Assumption 2 presents whether *p* should be an attachment to the core *c* or not.

**Assumption** **A2.** A external boundary protein p is affirmed as an attachment to the complex core c if satisfying cf(c+{p})>cf(c).

From the above assumption, the external boundary protein *p* improves the closeness function cf of the current cluster selected as an attachment. Through appending some attachment proteins to the current core, the topology of core can still be reserved, and thus all the proteins in each final predicted complex are densely connected and sparsely connected to the outside. Algorithm 3 is the pseudo code description.

**Algorithm 3:** Attach-proteins screening algorithm.**Require:** Protein complex cores CS. **Ensure:** The predicted complexes Complexes.
1:Complexes={};2:**for** each *c* in CS **do**3: **while** exists $\max_{v\in Neighbors(c)}\mathrm{cf}\left(c\cup \left\{v\right\}\right)>\mathrm{cf}\left(c\right)\;\;$ **do**4:  $c=c\cup \{\arg\max_{v\in Neighbors(c)}\mathrm{cf}\left(c\cup \left\{v\right\}\right)\};$
5: **end while**6: Complexes=Complexes∪{c};7:**end for**8:**return**Complexes;


### 4.3. Data Sources

Three publicly available yeast PPI networks, namely the Database of Interacting Proteins (DIP) data [30], Gavin data [14] and Srihari data collected by Srihari et al. [31], are used to evaluate the performance of our method CFOCM in protein complex prediction. DIP consists of 17,203 PPIs involving 4930 proteins, while Gavin data contains fewer proteins but is more densely connected, which consists of 6531 high-quality interactions among 1430 proteins. Srihiri data contains 20,000 interactions covering 3680 proteins derived from the BioGRID, IntAct, and MINT repositories.

To validate our predicted complexes, three reference sets of real complexes, denoted as the Munich Information Center for Protein Sequence (MIPS) [32], Saccharomyces Genome Database (SGD) [33], and CYC2008 [34], are selected as benchmarks. MIPS consists of 203 protein complexes manually curated from the literature, SGD contains 323 complexes derived from Gene Ontology-based complex annotations, and CYC2008 consists of 408 hand-curated complexes reliably backed by small-scale experiments.

The yeast GO annotation dataset is downloaded from the SGD database, and the submission data is February 2014.

## 5. Conclusions

In this paper, we have proposed a novel algorithm CFOCM for protein complex identification from the protein–protein interaction network. According to the fact that there some proteins involved in more than one biological function or cellular processes, CFOCM implements allowing overlaps between detected complexes. Meanwhile, CFOCM also takes into account the inherent core–attachment structure in the protein complexes. Moreover, CFOCM ensures topological similarity and functional interdependence between each pair of proteins within detected cores.

Comparison experiments between CFOCM and the other six state-of-the-art methods are carried out in DIP networks, Gavin networks and Srihari data, and the results of all tests show that CFOCM significantly outperforms the others. Moreover, CFOCM has been demonstrated to be capable of filtering the low-confidence or biological insignificant protein complexes via comparing with unCFOCM without consideration that the proteins in a complex core should occupy some common functions. In a word, CFOCM is efficient, robust, and it is applicable for helping biologists search for new biological meaningful protein complexes.

The follow-up works are ongoing. For instance, since some proteins still have not been functionally annotated, and we intend to find a more suitable strategy to handle this data problem, and design a parallel version of CFOCM to accelerate the operating speed. In addition, how to extend CFOCM to detect protein complexes and functional modules in dynamic PPI networks, which can be constructed by incorporating gene expression data, is also a promising direction.

## Figures and Tables

**Table 1:** Results of various approaches using DIP data.

Algorithms	MCL	MCODE	RNSC	COER	ClusterONE	COACH	CFOCM
# complexes	4838	63	543	592	341	746	748
$N_{p}$ (MIPS)	305	31	65	78	69	179	205
$N_{b}$ (MIPS)	117	42	96	113	89	134	126
$N_{p}$ (SGD)	621	39	106	117	112	231	285
$N_{b}$ (SGD)	262	53	134	138	121	176	168
$N_{p}$ (CYC2008)	853	46	134	153	145	311	351
$N_{b}$ (CYC2008)	358	55	149	168	132	215	196

**Table 2:** Results of various approaches using Gavin data.

Algorithms	MCL	MCODE	RNSC	COER	ClusterONE	COACH	CFOCM
# complexes	232	69	476	267	292	326	453
$N_{p}$ (MIPS)	59	31	22	69	65	106	191
$N_{b}$ (MIPS)	96	47	21	98	80	94	91
$N_{p}$ (SGD)	86	46	53	101	109	130	250
$N_{b}$ (SGD)	114	61	55	120	121	118	119
$N_{p}$ (CYC2008)	115	51	68	130	136	171	305
$N_{b}$ (CYC2008)	142	63	79	148	143	135	131

**Table 3:** Results of various approaches using Srihari data.

Algorithms	MCL	MCODE	RNSC	COER	ClusterONE	COACH	CFOCM
# complexes	4732	88	552	525	773	726	758
$N_{p}$ (MIPS)	325	26	78	92	117	219	225
$N_{b}$ (MIPS)	168	42	102	111	131	150	152
$N_{p}$ (SGD)	654	36	108	176	224	299	322
$N_{b}$ (SGD)	292	44	184	189	217	231	240
$N_{p}$ (CYC2008)	846	46	138	218	275	397	452
$N_{b}$ (CYC2008)	362	57	154	236	272	281	290

**Table 4:** Results of CFOCM and CFOCM without using Gene Ontology (GO) (unCFOCM) on DIP data.

Algorithms + Benchmark	# Complexes	$N_{p}$	$N_{b}$	Precision	Recall	F-Measure
CFOCM + MIPS	748	205	126	0.2741	0.6207	0.3802
unCFOCM + MIPS	862	213	130	0.2471	0.6404	0.3566
CFOCM + SGD	748	285	168	0.381	0.5201	0.4398
unCFOCM + SGD	862	297	175	0.3445	0.5418	0.4212
CFOCM + CYC2008	748	351	196	0.4693	0.4804	0.4748
unCFOCM + CYC2008	862	363	201	0.4211	0.4926	0.4541

**Table 5:** Results of CFOCM and CFOCM without using Gene Ontology (GO) (unCFOCM) on Gavin data.

Algorithms + Benchmark	# Complexes	$N_{p}$	$N_{b}$	Precision	Recall	F-Measure
CFOCM + MIPS	453	191	91	0.4216	0.4483	0.4345
unCFOCM + MIPS	551	197	92	0.3575	0.4532	0.3997
CFOCM + SGD	453	250	119	0.5519	0.3684	0.4419
unCFOCM + SGD	551	262	124	0.4755	0.3839	0.4248
CFOCM + CYC2008	453	305	131	0.6733	0.3211	0.4348
unCFOCM + CYC2008	551	321	138	0.5826	0.3382	0.4280

**Table 6:** Three protein-protein interaction (PPI) networks used in the experiments.

Dataset	#Proteins	#Interactions	Average Node Degree
DIP	4930	17203	6.98
Gavin	1430	6531	9.13
Srihari	3680	20,000	10.87
